# Advances, Implications, and Limitations of Low-Crude-Protein Diets in Pig Production

**DOI:** 10.3390/ani12243478

**Published:** 2022-12-09

**Authors:** Gabriel Cipriano Rocha, Marcos Elias Duarte, Sung Woo Kim

**Affiliations:** 1 Department of Animal Science, Universidade Federal de Viçosa, Viçosa 36570-900, MG, Brazil; 2 Department of Animal Science, North Carolina State University, Raleigh, NC 27695, USA

**Keywords:** essential amino acids, functional amino acids, growth performance, lysine, non-essential amino acids, pigs

## Abstract

**Simple Summary:**

Currently, five crystalline essential amino acids (lysine, methionine, threonine, tryptophan, and valine) are generally used, allowing animal nutritionists to formulate diets with low crude protein levels. Moreover, isoleucine may also be used depending on its economic value and the specific feeding program. Experimentally, it has been shown that further reduced crude protein levels can be achieved by supplemental histidine, leucine, and phenylalanine to the diets. However, decreasing the dietary crude protein level while maintaining optimal ratios of amino acids has shown contradictory effects on pigs’ growth performance. Due to the divergence in the literature and the importance for practical formulation strategies in the swine industry, a literature review and a meta-analysis were performed to estimate the minimum crude protein level that would not compromise pig performance. Based on the present review, there is a minimum crude protein level after which the growth performance of pigs can be compromised, even though diets are balanced for all essential amino acids. Considering average daily gain and the gain-to-feed ratio, respectively, these levels were estimated to be 18.4% and 18.3% crude protein for nursery, 16.1% and 16.3% crude protein for growing, and 11.6% and 11.4% crude protein for finishing pigs.

**Abstract:**

Currently, five crystalline essential amino acids (Lys, Met, Thr, Trp, and Val) are generally used, allowing formulation of low-crude-protein (CP) diets. Moreover, Ile may also be used depending on its economic value and the specific feeding program. Experimentally, it has been shown that further reduced CP levels can be achieved by supplemental His, Leu, and Phe to the diets. However, decreasing the dietary CP level while maintaining optimal ratios of amino acids has shown contradictory effects on pigs’ growth performance. Due to the divergence in the literature and the importance for practical formulation strategies in the swine industry, a literature review and a meta-analysis were performed to estimate the minimum CP level that would not compromise pig performance. Based on the present review, there is a minimum CP level after which the growth performance of pigs can be compromised, even though diets are balanced for essential amino acids. Considering average daily gain and gain to feed, respectively, these levels were estimated to be 18.4% CP (95% confidence interval [CI]: 16.3 to 18.4) and 18.3% CP (95% CI: 17.4 to 19.2) for nursery, 16.1% CP (95% CI: 16.0 to 16.2) and 16.3% CP (95% CI: 14.5 to 18.0) for growing, and 11.6% CP (95% CI: 10.8 to 12.3) and 11.4% CP (95% CI: 10.3 to 12.5) for finishing pigs.

## 1. Introduction

The feeding programs for commercially raised pigs can be broken down into nursery, growing, and finishing categories [1,2,3]. For each category, the total dietary supply of crude protein (CP) must be sufficient to provide the requirement for essential amino acids (EAA) as well as the necessary nitrogen required for the synthesis of non-essential amino acids (NEAA) [4,5]. Thus, it is generally accepted that requirements are predominately based on intake of a complete set of amino acids instead of CP [1,6]. However, decreasing the dietary CP level while maintaining optimal ratios of amino acids has shown contradictory effects on growth performance in pigs [7,8,9].

Currently, five crystalline essential amino acids (Lys, Met, Thr, Trp, and Val) are generally used from the nursery through finishing phases, allowing formulation of low-CP diets. Moreover, Ile may also be used depending on its economic value and the specific feeding program. Experimentally, it has been shown that further reduced CP levels can be achieved by supplemental His, Leu, and Phe to the diets. However, the resulting lower-CP diet consequently decreases the total dietary NEAA, intact protein, and the related release of bioactive compounds such as peptides and isoflavones, which may impair the growth performance of pigs [10,11,12].

Concerning both economic and environmental perspectives, the reduction of dietary CP coupled with supplementation of crystalline amino acids is an effective strategy for the swine industry to reduce cost and pollution [13,14]. Although the economic efficiency of low-CP diets may vary with the price of ingredients and pig performance [15,16], the benefits for the environment due to lower nitrogen excretion are well established [17,18].

Notably, low-CP diets would be easily accepted by the swine industry if pig performance is not reduced. Unfortunately, some experiments have shown that even with the supplementation of EAA, low-CP diets may impair growth performance [8,12,19]. Moreover, under commercial conditions, CP and not amino acids may be limiting performance capabilities [6]. Thus, the focus of the present review was to better understand the advances and implications of reducing CP in diets balanced for all EAA and its associated effect on growth performance in pigs. Based on the literature review, a meta-analysis was performed to estimate the minimum CP level that would not compromise pig growth performance.

## 2. Meta-Analysis

In this review, a database was constructed from indexed publications that addressed the effect of dietary CP level on the growth performance of pigs. Data from peer-reviewed papers were obtained from different digital databases (Google Scholar, Science Direct, Scopus, Scielo, and PubMed). The prospective papers were then selected based on specific criteria to evaluate their inclusion or exclusion from the present review. The main criteria for the selection of papers were that they (1) evaluated different CP levels; (2) had similar standardized ileal digestible (SID) Lys and metabolizable energy (ME) between control and low-CP treatments; (3) had all EAA in experimental diets meeting or exceeding the EAA-to-Lys ratio (NRC, 2012) (when not presented, it was estimated after reformulating the diets based on the NRC [1]); and (4) clearly reported performance responses. There were 46 papers that fulfilled all selection criteria. Data from papers were subsequently divided into three groups: nursery (17 papers with 23 experiments); growing (17 papers with 24 experiments); and finishing (12 papers with 13 experiments). Within each selected study, the respective data of each CP level were entered in a separate row. Similarly, if more than one experiment (or phase) was reported in the same study, the data of each experiment were entered separately as different studies.

The NLIMIXED procedure of SAS (SAS. Inc., Cary, NC, USA) was used to conduct the meta-analysis using a nonlinear model [20,21,22]. The meta-analysis models included the levels of CP, SID Lys:CP ratio, and L-lysine supplementation as the independent variables (X axis) and the average daily feed intake (ADFI), average daily gain (ADG), and gain to feed (G:F) as dependent variables (Y axis). The studies were used in the model as random component for the asymptote. The broken-line model used for the meta-analysis was: Y = a + b × (R − X) + STU + e. Where ‘Y’ is the dependent variable; ‘a’ is the asymptote; ‘b’ is the slope; ‘X’ is the independent variable; ‘R’ is the breakpoint in which ‘(R − X)’ is defined as zero when X ≥ R for variable CP and ‘(R − X)’ is defined as zero when X ≤ R for variables L-lysine, and SID Lys:CP; STU is the random effect of studies; and ‘e’ is a the residual error.

The fitted models were selected when *p* < 0.05 for the asymptote, slope, and breakpoint. When more than one model (linear or broken-line) was fitted with the same variables, the greater R^2^ and the least 2-log likelihood were defined as the criteria to select the best fitting model. The broken-line models had the greater R^2^ and the least 2-log likelihood compared with linear models. It was not possible to fit a model (linear or broken-line) for ADFI considering the independent variables (X axis) used, levels of CP, SID Lys:CP ratio, and L-lysine.

## 3. Low-CP Formulations

### 3.1. Nursery Phase

According to the NRC [1] and the Brazilian Tables [3], a corn- soybean meal (SBM) diet for 7 kg pigs contain around 20.5% CP. At this CP level containing 1.35% SID Lys, at least five EAA (Lys, Met, Thr, Trp, and Val) must be supplemented to achieve the amino acids requirements for nursery pigs based on the ideal protein concept. In line, lower-CP diets could be formulated with the addition of others EAA to effectively meet the amino acid requirements.

A CP level of 17.0% or lower supplemented with crystalline amino acid was not sufficient to maintain the growth performance of nursery pigs [23,24,25]. However, in those studies, the next limiting amino acid was not appropriately supplied in the diet. For example, Deng et al. [24] and Spring et al. [25] supplemented low-CP diets with Lys, Met, Thr, Trp, and branched chain amino acids (BCAA). Still, the calculated ratio of His and Phe to Lys on their lower-CP level diets were below the requirements suggested by NRC [1], and thus those EAA may have limited the pig performance.

In collaboration, some studies showed that reducing CP content from approximately 20.0 to 17.0% without balancing the amino acids impaired the growth of nursery pigs, which was restored to the level of the control after supplementation with BCAA and maintaining the proper ratio of His and Phe to Lys [26,27]. Other studies have also proposed that dietary CP could be reduced to approximately 17.0% if the concentrations of EAA were maintained within the recommended range through the supplementation of amino acids [28,29,30].

Low-CP diets balanced with EAA have been used as part of an overall strategy to maintain intestinal health in pigs [23,31]. Low-CP diets have lower indigestible carbohydrates (stachyose and raffinose) and antigenic protein (glycinin and β-conglycinin), which are considered antinutritional factors [32,33]. Moreover, it may reduce proliferation of pathogenic bacteria and their potential toxins to the gastrointestinal tract such as ammonia, polyamine, and others [34]. Altogether, low-CP diets result in improved growth performance of nursery pigs through enhancing intestinal health, as demonstrated by Zhao et al. [31], when CP was reduced from 22.5 to 18.5%.

On the other hand, Yue and Qiao [35] reported detrimental effects on ADG and ADFI of pigs when evaluating the reduction of CP (from 23.1 to 17.2%), even with the inclusion of Val, Ile, His, and Phe. Gloaguen et al. [36] evaluated low-CP diets with supplemental EAA and performance was still reduced when pigs were fed 14.0 and 12.7% CP diets compared to 19.7 and 16.8% CP diets. Similarly, Millet et al. [6] found that decreasing CP levels (from 19.0 to 14.0%) linearly decreased ADG, even with supplementation of BCAA, His, Phe, and Tyr. The former authors suggested that not amino acids but rather the CP may be limiting the growth performance in lower-CP diets.

In fact, it has been suggested that the generation of NEAA from EAA may become a limiting factor for normal growth performance in pigs fed low-CP diets [11,36]. Nursery pigs fed a lower-CP diet present a reduction in blood concentration of NEAA, in particular, Arg, Gln, Glu, and Pro [24,35,37]. Thus, some of the NEAA can become functionally deficient in low-CP diets and their supplementation may be necessary to maintain the health and growth of nursery pigs. Considering the functional roles of selected NEAA [38,39], lack of Arg and Gln could cause negative impacts on the health, intestinal development, and growth of nursery pigs [40,41,42,43]. Moreover, lower-CP diets supplemented with EAA may have a deficiency of intact proteins and the related bioactive peptides [10,24]. In light of this, a drastic reduction in CP must be accompanied by other precautions, such as observing the relationship between EAA and NEAA, total nitrogen in the diet, and possible lack of bioactive peptides, nucleotides, and non-nitrogen compounds, including isoflavones [6,10,44].

Recently, Batson et al. [19] observed decreased ADG in pigs fed 18.0% CP diets compared with 21.0% CP diets, although all EAA were balanced in the diets. Interestingly, these authors and others [9,35,45,46] reported that low-CP diets improved gut health and fecal consistency of nursery pigs, while still presenting depressed growth performance. The resulting impairment of growth may be related to the reduction of protein synthesis in multiple tissues, such as the liver, pancreas, and longissimus muscle in pigs fed low-CP diets [24]. It has been suggested that NEAA and peptides may increase lean deposition through the activation of the protein synthesis pathway [47,48] and may partially explain the lower growth performance observed by those authors in the low-CP diets.

**Figure 1:**
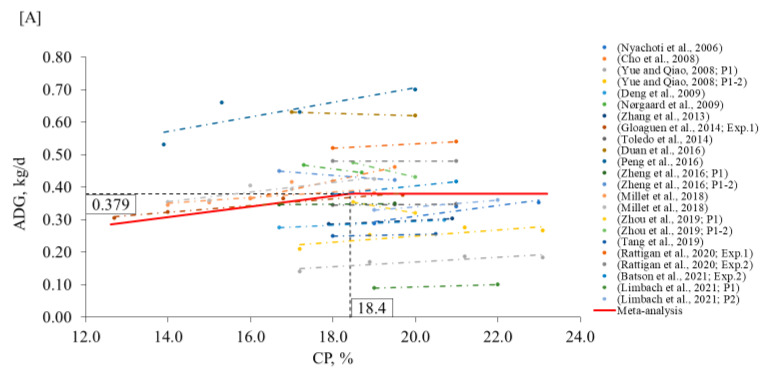

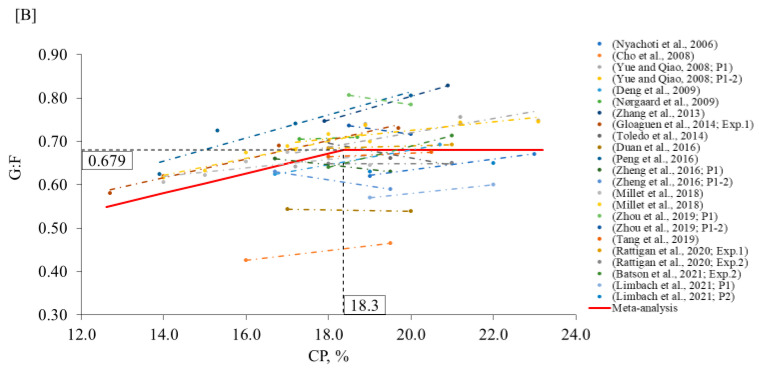
Changes in ADG (**A**) and G:F (**B**) in response to dietary CP in the nursery phase using a broken-line analysis. The equation for ADG was ADG = 0.379 − 0.016 × zl (R^2^ = 0.98) and the breakpoint was CP level at 18.4% (95% CI: 16.3–18.4) when ADG was 0.379 kg/d. The *p*-value for the asymptote was <0.001, for the slope it was <0.001, and for the breaking point it was <0.001. The equation for G:F was G:F = 0.679 − 0.023 × z1 (R^2^ = 0.91) and breakpoint was CP level at 18.3% (95% CI: 17.4–19.2) when G:F was 0.679. The *p*-value for the asymptote was <0.001, for the slope it was <0.001, and for the breaking point it was <0.001. Where, if CP is ≥breakpoint, then z1 = 0; if CP is <breakpoint, then z1 = CP − breakpoint [6,9,11,19,23,24,26,27,29,30,35,36,37,49,50,51,52].

Due to the divergence in the literature and the importance and implications for practical formulation strategies on the swine industry, a meta-analysis was performed to estimate the minimum CP level that would not compromise pig performance. The model estimated 18.4% CP (95% confidence interval [CI]: 16.3–18.4) as the breakpoint below which the ADG would be reduced (Figure 1A; Table 1). When considering the G:F, the estimated level was 18.3% CP (95% CI: 17.4–19.2) (Figure 1B). The proposed minimum CP level for nursery diets is below the level presented in the NRC [1] and the Brazilian Tables [3], indicating that lower CP levels than those proposed in the tables might be used to formulate nursery diets.

**Figure 2:**
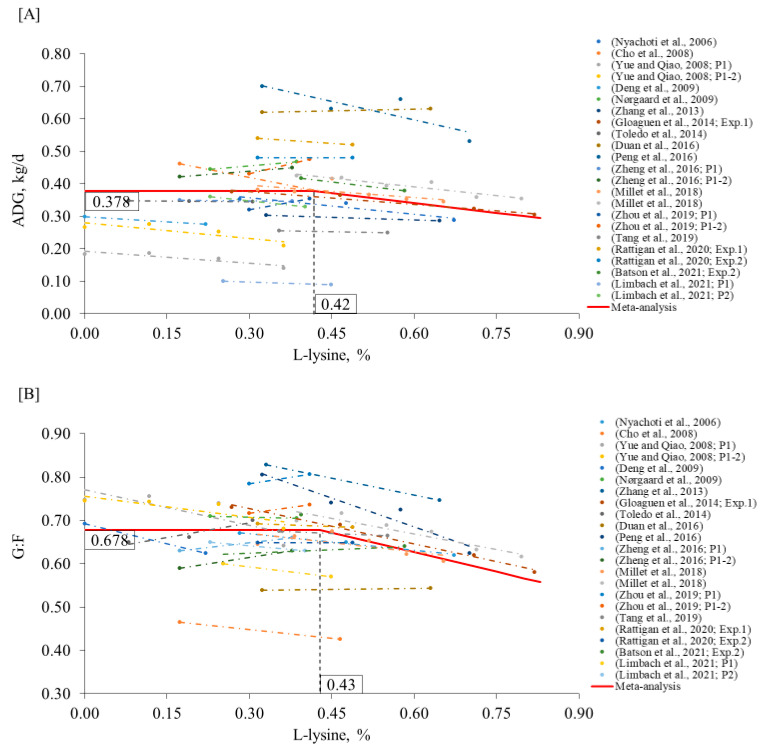
Changes in ADG (**A**) and G:F (**B**) in response to L-lysine supplementation in the nursery phase using a broken-line analysis. The L-lysine axis is based on 100% purity, calculated from L-lysine HCl (78.8% purity) or L-lysine sulfate (60.0% purity). The equation for ADG was ADG = 0.378 + 0.202 × zl (R^2^ = 0.97) and the breakpoint was L-lysine level at 0.42% (95% CI: 0.30–0.53) when ADG was 0.378 kg/d. The *p*-value for the asymptote was <0.001, for the slope it was <0.001, and for the breaking point it was <0.001. The equation for G:F was G:F = 0.678 + 0.301 × z1 (R^2^ = 0.91) and breakpoint was CP level at 0.43% (95% CI: 0.35–0.50) when G:F was 0.678. The *p*-value for the asymptote was <0.001, for the slope it was <0.001, and for the breaking point it was <0.001. Where, if L-lysine is ≤breakpoint, then z1 = 0; if L-lysine is >breakpoint, then z1 = L-lysine − breakpoint [6,9,11,19,23,24,26,27,29,30,35,36,37,49,50,51,52].

Additionally, we also estimated the higher levels of crystalline lysine that could be supplemented before compromising ADG and G:F, which were 0.42% (95% CI: 0.30–0.53) and 0.43% (95% CI: 0.35–0.50), (Figure 2A,B), respectively. Using an average of 0.425%, this is equivalent to 0.54% of L-lysine HCl (78.8% purity) or 0.71% of L-lysine sulfate (60.0% purity). To our knowledge, this is the first study to propose a maximum level of crystalline lysine supplementation and thus could be used as a starting point for future studies.

**Figure 3:**
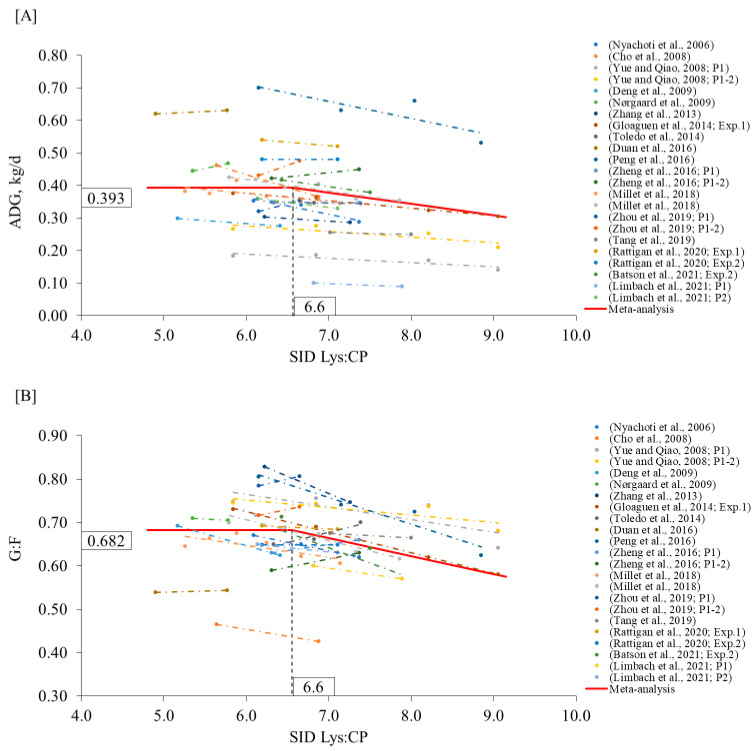
Changes in ADG (**A**) and G:F (**B**) in response to the ratio between standard ilea digestible Lys to crude protein (SID Lys:CP) in the nursery phase using a broken-line analysis. The equation for ADG was ADG = 0.393 + 0.035 × zl (R^2^ = 0.98) and the breakpoint was SID Lys:CP ratio at 6.6% (95% CI: 5.9–7.2) when ADG was 0.393 kg/d. The *p*-value for the asymptote was <0.001, for the slope it was <0.001, and for the breaking point it was <0.001. The equation for G:F was G:F = 0.682 + 0.041 × z1 (R^2^ = 0.91) and breakpoint was SID Lys:CP ratio at 6.6% (95% CI: 6.1–7.0 ) when G:F was 0.682. The *p*-value for the asymptote was <0.001, for the slope it was <0.001, and for the breaking point it was <0.001. Where, if SID Lys:CP is ≤breakpoint, then z1 = 0; if SID Lys:CP is >breakpoint, then z1 = SID Lys:CP − breakpoint. [6,9,11,19,23,24,26,27,29,30,35,36,37,49,50,51,52].

The use of a SID Lys:CP ratio may also prove to be a good estimator of to what extent CP could be lowered [6,53,54]. In this way, it was obtained that 6.6% (95% CI: 5.9–7.2) of SID Lys:CP is the breakpoint above which the ADG is compromised (Figure 3A). When considering the G:F, the estimated level was the same, 6.6% (95% CI: 6.1–7.0) (Figure 3B). These levels are quite similar to the 6.4% SIDLys:CP proposed by Millet et al. [6]. The use of the SID Lys:CP can be used as a reference to maintain sufficient EAA to meet the pigs’ need for NEAA.

In summary, low-CP diets should be formulated by assuming a minimum CP level to avoid the limitation of other nutrients that may be deficient when reducing the CP below a certain level. Based on the present review, the minimum CP level is 18.3% for nursery diets with the concentration of EAA being maintained as recommended (NRC, 2012). Moreover, 0.54% of L-lysine HCl or 0.71% of L-lysine sulfate were estimated to be the maximum supplementation levels above which growth performance might be compromised. Finally, 6.6% SID Lys:CP was estimated to be the breakpoint for growth performance.

### 3.2. Growing Phase

Compared to the nursery, growing phase diets are formulated to a lower CP level. At the beginning of the growing phase, a corn-SBM diet with 17.5% CP could be formulated, supplementing only Lys, Met, Tre, and Trp to achieve the suggested amino acid requirements [1,3]. Lower CP levels can be obtained with the additional supplementation of the next limiting amino acids.

Madrid et al. [55] demonstrated that diets formulated using the ideal protein concept and supplemented with amino acids did not affect performance when dietary CP decreased from 16.0 to 14.0%. Supplementing all the EAA except Leu, Qiu et al. [56] demonstrated that CP could be decreased from 18.0 to 14.0% without compromising growth performance. According to Powell et al. [57], the growth performance of pigs was maintained when the dietary CP level was reduced from 18.2 to 13.4% while keeping the proper ratio of amino acids. In agreement, Zhao et al. [14] also found that a reduction in dietary CP from 17.4 to 13.5% with all EAAs balanced had no negative effect on pig growth performance.

Contrary to the previous studies, Li et al. [58], showed that decreasing the CP level from 18.3% to 15.1% resulted in decreased ADG and G:F although the calculated ratio of all EAA to Lys was in agreement with the NRC [1]. In this way, others have balanced diets for all the EAA but also failed to lower CP while maintaining similar growth performance. For example, Peng et al. [11] showed that pig growth performance was similar when dietary CP level was reduced from 20.0 to 15.3%; however, a further decrease to 13.9% resulted in lower ADG and G:F compared with pigs receiving a 20.0% CP diet. According to Che et al. [8], a moderate reduction in CP from 16.7 to 14.7% maintained the growth performance of pigs; however, a reduction to 12.9% markedly decreased growth performance. Additionally, Roux et al. [59] also failed to show similar growth performance with the supplementation of EAA in low-CP diets (18.2 vs. 13.4%).

The discrepancies among studies could be due to the variation in CP levels designed for the positive control diets [8]. A dietary CP level of 16.0 or 20.0% as a positive control are different concerning intact protein, peptides, and NEAA content. In this way, a further reduction of CP level may differentially affect animal growth performance. Similarly, Peng et al. [11] and Lynegaard et al. [46] also report that their positive control diets were already a lower CP level for pigs, and therefore, the lower-CP treatments may have compromised the growth performance of growing pigs. Moreover, it has been suggested that the energy content of SBM proposed in the tables [1,3] might be underestimated [60,61]. Thus, the ME of SBM can also affect the results of studies aiming at lower CP levels.

**Figure 4:**
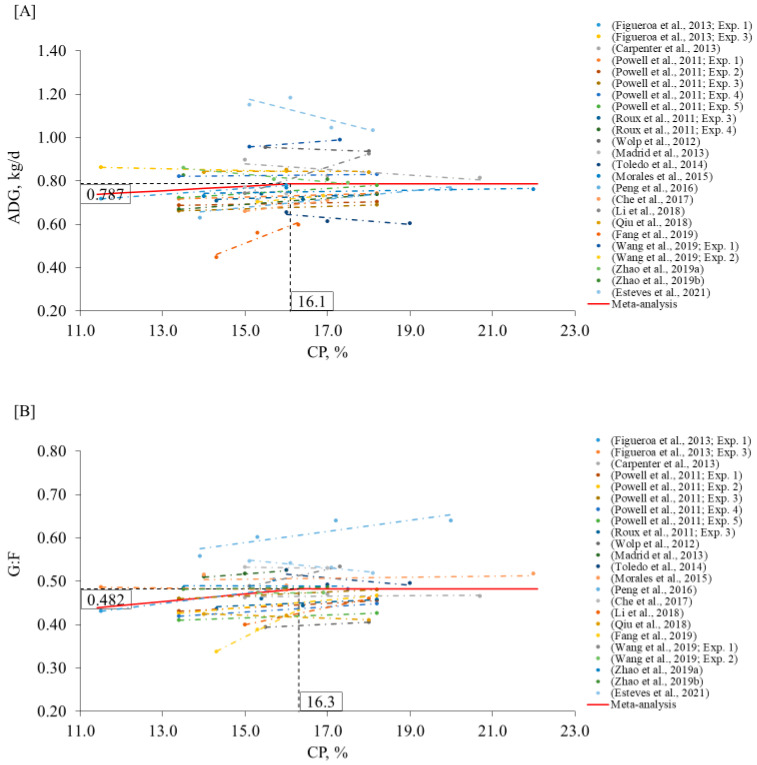
Changes in ADG (**A**) and G:F (**B**) in response to dietary CP in the growing phase using a broken-line analysis. The equation for ADG was ADG = 0.787 − 0.010 × zl (R2 = 0.93) and the breakpoint was CP level at 16.1% (95% CI: 16.0–16.2) when ADG was 0.787 kg/d. The *p*-value for the asymptote was <0.001, for the slope it was 0.050, and for the breaking point it was <0.001. The equation for G:F was G:F = 0.482 − 0.008 × z1 (R2 = 0.93) and breakpoint was CP level at 16.3% (95% CI: 14.5–18.0) when G:F was 0.482. The *p*-value for the asymptote was <0.001, for the slope it was 0.008, and for the breaking point it was <0.001. Where, if CP is ≥breakpoint, then z1 = 0; if CP is <breakpoint, then z1 = CP − breakpoint [7,8,11,13,14,18,55,56,57,58,59,62,63,64,65,66,67].

Similar to the nursery phase, the extent to which the CP content of the diets can be reduced without affecting the growth performance has been conflicting in the growing phase. Based on the model from the meta-analysis, CP content can be decreased to 16.1% (95% CI: 16.0–16.2) and 16.3% (95% CI: 14.5–18.0) when formulating for optimal ADG and G:F, as long as EAA meet the requirements of pigs (Figure 4A,B). For a similar phase, the NRC [1] and Brazilian Tables [3] suggest 15.7% and 18.9% CP, respectively. The discrepancies among CP levels could be due to the variation in genotypes as the efficiency of utilization of dietary protein for body protein deposition is strongly related to pig genotype [1,3]. The present results are in between those suggestions. However, when below those levels of CP, other factors should be considered, such as the level of other nutrients (NEAA, peptides, and others).

For the growing phase, it was not possible to estimate a breakpoint for L-lysine supplementation on growth performance. However, the average maximum level of L-lysine supplementation in the growing trials was 0.42 ± 0.14% (Table 2), which is equivalent to 0.53% of L-lysine HCl (78,8% purity) or 0.70% of L-lysine sulfate (60% purity). This maximum level of feed-grade lysine supplementation is quite similar to the level estimated for the nursery phase. Over-feeding crystalline lysine and other amino acids can be costly, and therefore, the knowledge of a maximum level above which performance is compromised is important.

Considering the studies in the meta-analysis, it was not possible to estimate a breakpoint for SID Lys:CP ratio on growth performance of growing pigs. As mentioned before, this is a relatively new estimator, recently proposed by Millet et al. [6] working with nursery pigs.

### 3.3. Finishing Phase

Protein is a relatively expensive nutrient and due to the increase in feed intake during the finishing phases, the efficiency of nutrient use greatly impacts the costs incurred by the production system [4]. An early-finishing (70 to 100 kg) diet may be formulated with approximately 12.3% CP and a late-finishing (100 kg to slaughter) diet with approximately 11.0% CP while maintaining the concentration of EAA as recommended [1,3].

In agreement, Xie et al. [68] and Zhou et al. [69] found that the CP levels could be reduced from 15.3 to 12.0% and from 14.1 to 10.1% CP, respectively, without affecting growth performance in the early-finishing phase. In addition to similar performance, Norgaard et al. [70] observed that a reduction in CP from 15.9 to 13.6% did not affect carcass characteristics. Qin et al. [17] also demonstrated no effect on the growth performance, carcass characteristics, and meat quality of late-finishing pigs when CP was reduced from 14.3 to 12.3%.

However, some studies evaluating low-CP diets have reported decreases in growth performance, even when diets are balanced for the limiting EAA. Li et al. [71] showed that decreasing the CP level from 16.3% to 13.2% resulted in decreased ADG and G:F. It is interesting and worthwhile to note that the lower-CP diet improved meat quality. Soto et al. [12] evaluated the effect of CP levels for early- (from 13.1 to 9.0% CP) and late- (from 12.9 to 8.9% CP) finishing pigs. They observed that reduction in CP negatively affected growth performance in both trials. However, carcass yield, backfat, and loin depth were not affected by CP levels. Comparing 16.0 and 12.0% CP diets, Zhou et al. [72] showed that pigs fed a low-CP diet had lower ADG and G:F, with no effect on body fat content.

Considering that all EAA were properly supplied in the cited papers, there are some explanations in the literature for the negative effects on growth when low-CP diets are fed. As previously mentioned, a deficiency of NEAA in low-CP diets may affect pig growth performance. Moreover, supplementing EAA leads to a reduction in the supplementation of protein sources, such as soybean meal. These ingredients contain biologically active compounds, such as isoflavones, saponins, and bioactive peptides, that may also be important to maintain growth performance [10,12,44]. Diet-derived peptides can exert actions at the level of the small intestine, with the resulting intestinally-generated signals having impacts on the whole body. Bioactive peptides have been suggested to possess antimicrobial, antioxidant, and immunomodulatory activities [73]. Furthermore, it is noteworthy that a significant amount of small peptides are absorbed intact [74,75], and the specificity of protein hydrolysis and the related release of peptides differ between feed ingredients, causing different effects on animal physiology [76]. Thus, reducing the protein sources to achieve a lower CP level may affect animal growth performance.

Apart from improvements in growth performance and carcass characteristics, the swine feed industry has been pushed to reduce dietary CP and simultaneously the excretion of nitrogen, which can contribute to greenhouse gas emissions [14,17,18]. In this way, it is well established the benefits of reduced CP diets on lowering the environmental impact of pig production [14,17,70]. However, the relationship between low CP and the environment was not the focus of the present review and may be found elsewhere [18,77]. Meanwhile, new research should continue to focus on strategies utilizing low-CP diets while not compromising growth performance and pig profitability.

Similar to the finishing phase, contradictory results have been reported when considering the entire growing to finishing phases. Working with growing-finishing pigs (25–110 kg) in two climatic conditions, Wang et al. [66] evaluated the effect of low and high CP diets. In both experiments, pigs fed low-CP diets supplemented with EAA had similar growth performance, carcass characteristics, and meat quality to pigs fed high CP diets. In the same way, Zhao et al. [14] showed no effects of decreasing CP in the diets on growth performance and carcass characteristics of growing-finishing pigs (25–125 kg). Recently, Le Dinh et al. [77] found that reducing CP with EAA supplementation increased ADG and ADFI but also reduced muscle thickness. Conversely, Fang et al. [65] found that growing/finishing pigs (30–105 kg) fed low-CP diets supplemented with EAA resulted in reduced growth performance.

**Figure 5:**
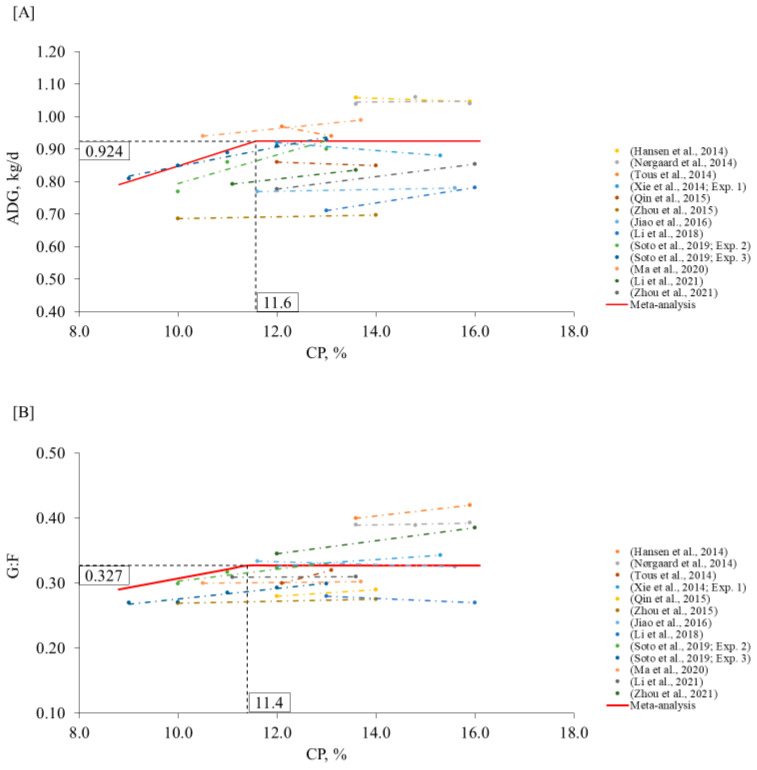
Changes in ADG (**A**) and G:F (**B**) in response to dietary CP in the finishing phase using a broken-line analysis. The equation for ADG was ADG = 0.924 − 0.048 × zl (R^2^ = 0.98) and the breakpoint was CP level at 11.6% (95% CI: 10.8–12.3) when ADG was 0.924 kg/d. The *p*-value for the asymptote was <0.001, for the slope it was 0.050, and for the breaking point it was <0.001. The equation for G:F was G:F = 0.327 − 0.014 × z1 (R^2^ = 0.96) and breakpoint was CP level at 11.4% (95% CI: 10.3–12.5) when G:F was 0.327. The *p*-value for the asymptote was <0.001, for the slope it was 0.006, and for the breaking point it was <0.001. Where, if CP is ≥breakpoint, then z1 = 0; if CP is <breakpoint, then z1 = CP − breakpoint [12,17,68,69,70,71,72,78,79,80,81,82].

In agreement, the model from the meta-analysis suggested that the CP level below 11.6% (95% CI: 10.8–12.3) and 11.4% (95% CI: 10.3–12.5) can compromise the ADG and G:F, respectively (Figure 5). These levels are in between the levels suggested in NRC [1] for early-(12.1% CP) and late-(10.5% CP) finishing pigs, probably because we considered both phases together in our model. On the other side, the proposed levels are below the average recommendations in Brazilian Tables [3] for early- (13.3% CP) and late- (11.9% CP) finishing pigs. Thus, CP levels lower than the Brazilian Tables [3] recommendations might be used without compromising pig growth performance.

**Figure 6:**
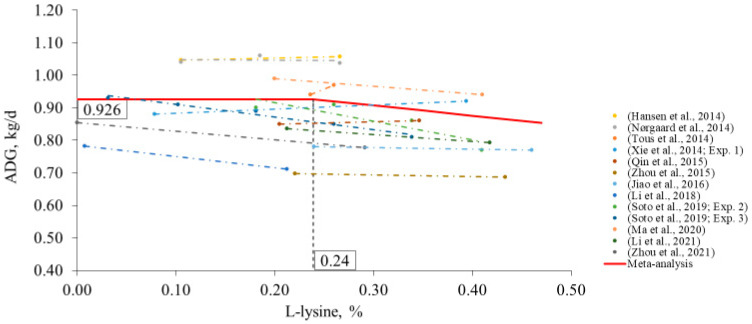
Changes in ADG in response to L-lysine supplementation in the finishing phase using a broken-line analysis. The L-lysine axis is based on 100% purity, calculated from L-lysine HCl (78.8% purity) or L-lysine sulfate (60.0% purity). The equation was ADG = 0.926 + 0.314 × zl (R^2^ = 0.97) and the breakpoint was L-lysine level at 0.24% (95% CI: 0.10–0.37) when ADG was 0.926 kg/d. The *p*-value for the asymptote was <0.001, for the slope it was 0.033, and for the breaking point it was 0.002. Where, if L-lysine is ≤breakpoint, then z1 = 0; if L-lysine is >breakpoint, then z1 = L-lysine − breakpoint [12,17,68,69,70,71,72,78,79,80,81,82].

Concerning feed grade lysine, the breakpoint for ADG was 0.24% L-lysine (95% CI: 0.10–0.37; Figure 6), which is equivalent to the supplementation of 0.30% L-lysine-HCl or 0.40% L-lysine sulfate. The proposed level of L-lysine is lower than that for nursery and growing phases because lysine supplementation in finishing diets is notably lower than in the initial phases (Table 3). Considering the studies in the meta-analysis, a breakpoint for G:F was not found (a model was not fit). Additionally, a breakpoint for SIDLys:CP ratio on growth performance was not found.

## 4. Conclusions

In conclusion, there is a minimum CP level after which the growth performance of pigs can be compromised, even though diets are balanced for all EAA. Apparently, there is a level after which other nutrients such as NEAA, bioactive compounds, and others become limiting. Based on the meta-analysis, considering the ADG and G:F the minimum levels of CP were estimated at 18.4% and 18.3% for nursery, 16.1% and 16.3% for growing, and 11.6% and 11.4% for finishing pigs, respectively. Moreover, it was estimated the higher levels of L-lysine (100% purity) to be supplemented before compromising growth performance were 0.42% for ADG and 0.43% for G:F in the nursery phase and 0.24% for ADG in the finishing phase. Additionally, a level of 0.42% L-lysine supplementation is suggested for growing pigs. Finally, it was obtained that 6.6% of SID Lys:CP is the ratio above which the ADG and G:F of nursery pigs are compromised. Considering that feed grade L-lysine HCl is 78% lysine, based on the meta-analysis, optimal allowances of the use of L-lysine HCl are 0.54% in feeds for nursery and growing pigs and 0.31% in feeds for finishing pigs. Applying these optimal levels of L-lysine HCl in swine feeds should not compromise the growth performance of pigs if the ideal ratios of lysine to other EAA and ME are considered.

## Figures and Tables

**Table 1:** Summary of the studies included in the meta-analysis to estimate changes in average daily gain and gain to feed in the nursery phase.

BW, kg	CP Level, %		SID Lys, %	Supplemental L-lysine ^1^, %	Additional Amino Acids ^2^	Reference ^3^
	Control	Lower				
6–12	23.0	19.0	1.40	0.67	Ile	Nyachoti et al. [23]
13–19	19.5	16.0	1.10	0.46	-	Cho et al. [49]
6–8	23.1	17.2	1.30	0.36	Val, Ile, His, Phe	Yue and Qiao [35] (P1)
6–10	23.1	17.2	1.30	0.36	Val, Ile, His, Phe	Yue and Qiao [35] (P1–2)
8–11	20.7	16.7	1.07	0.22	Val, Ile, Leu	Deng et al. [24]
9–21	18.7	17.3	1.00	0.39	Val, Ile	Nørgaard et al. [26]
8–11 *	20.9	17.9	1.30	0.65	Val, Ile, Leu	Zhang et al. [37]
12–16	19.7	12.7	1.15	0.82	Val, Ile, His, Phe, Leu	Gloaguen et al. [36] (Exp.1)
6–16	21.0	18.0	1.33	0.31	Val, Ile	Toledo et al. [50]
9–36	20.0	17.0	0.98 *	0.63	Val, Ile, Leu	Duan et al. [29]
13–18	20.0	13.9	1.23	0.70	Val, Ile, His, Phe, Leu	Peng et al. [11]
8–9	19.5	16.7	1.23	0.38	Val, Ile, Leu	Zheng et al. [27] (P1)
8–18	19.5	16.7	1.23	0.38	Val, Ile, Leu	Zheng et al. [27] (P2)
8–21 *	19.0	14.0	1.00	0.65	Val, Ile, His, Phe, Leu, Tyr	Millet et al. [6]
8–22 *	19.0	14.0	1.10	0.80	Val, Ile, His, Phe, Leu, Tyr	Millet et al. [6]
8–13	20.0	18.5	1.23	0.41	Val, Ile	Zhou et al. [51] (P1)
8–21	20.0	18.5	1.23	0.41	Val, Ile	Zhou et al. [51] (P1–2)
7–11	20.5	18.0	1.43	0.55	Val	Tang et al. [52]
6–25 *	21.0	18.0	1.29	0.49	Val	Rattigan et al. [30] (Exp.1)
7–24 *	21.0	18.0	1.29	0.49	Val	Rattigan et al. [30] (Exp.2)
6–12	21.0	18.0	1.40	0.58	Val, Ile, His	Batson et al. [19] (Exp.2)
5–7	22.0	19.0	1.50 *	0.45	Ile, His, Phe	Limbach et al. [9] (P1)
5–12	22.0	19.0	1.35 *	0.40	His, Phe	Limbach et al. [9] (P2)

^1^ Supplemented feed grade L-lysine based on the purity of L-lysine HCl or L-lysine sulfate in the lower-CP diet. ^2^ Additional supplemented feed grade amino acids in low-CP diets in addition to Lys, Met, Tre, and Trp. ^3^ References listed in year order, only studies and treatments balanced for all essential amino acids were used. When necessary, amino acids were estimated after reformulating the diets [1]. * Estimated levels.

**Table 2:** Summary of the studies included in the meta-analysis to estimate changes in average daily gain and gain to feed in the growing phase.

BW, kg	CP Level, %		SID Lys, %	Supplemental L-lysine ^1^, %	Additional Amino Acids ^2^	Reference ^3^
	Control	Lower				
20–46 *	16.0	12.0	0.77 *	0.45	Val, Ile	Figueroa et al. [7] (Exp. 1)
21–46 *	16.0	12.0	0.77 *	0.45	Val, Ile	Figueroa et al. [7] (Exp. 3)
44–65 *	20.7	15.0	1.03 *	0.49	-	Carpenter et al. [13]
19–39	18.2	13.4	0.83	0.33	Val, Ile	Powell et al. [57] (Exp. 1)
21–40	18.2	13.4	0.83	0.33	Val, Ile	Powell et al. [57] (Exp. 2)
19–38	18.2	13.4	0.83	0.33	Val, Ile	Powell et al. [57] (Exp. 3)
23–45	18.2	13.4	0.83	0.33	Val, Ile	Powell et al. [57] (Exp. 4)
23–44	18.2	13.4	0.83	0.33	Val, Ile	Powell et al. [57] (Exp. 5)
24–44	18.2	14.3	0.83	0.26	-	Roux et al. [59] (Exp. 3)
20–40	18.2	13.4	0.83	0.33	Val	Roux et al. [59] (Exp. 4)
37–65 *	18.0	15.5	0.90	0.17	-	Wolp et al. [62]
23–59	16.0	14.0	0.83	0.46	-	Madrid et al. [55]
15–30	16.0	14.0	1.14	0.36	Val, Ile	Toledo et al. [63]
24–40	22.0	14.0	0.98	0.63	Val, Ile, His, Phe, Leu	Morales et al. [64]
14–34	20.0	13.9	1.23	0.70	Val, Ile, His, Phe, Leu	Peng et al. [11]
16–33	17.0	15.0	1.23	0.62	Val, Ile, His, Phe, Leu	Che et al. [8]
36–60	18.3	15.2	0.96	0.36	-	Li et al. [58]
30–67	18.0	14.0	0.98	0.50	Val	Qiu et al. [56]
31–42	16.3	14.3	0.83 *	0.16	-	Fang et al. [65]
29–63	17.0	15.0	1.01	0.54	Val	Wang et al. [66] (Exp. 1)
25–60	17.0	15.0	0.98	0.47	Val	Wang et al. [66] (Exp. 2)
25–51	17.5	13.5	0.98	0.54	Val, Ile, His, Phe	Zhao et al. [67]
24–53	17.0	13.5	0.98	0.39	Val, Ile, His, Phe	Zhao et al. [14]
30–50	18.1	15.1	1.07	0.47	Val, Ile	Esteves et al. [18]

^1^ Supplemented feed grade L-lysine based on the purity of L-lysine HCl or L-lysine sulfate in the lower-CP diet. ^2^ Additional supplemented feed grade amino acids in low-CP diets in addition to Lys, Met, Tre, and Trp. ^3^ References listed in year order, and only studies and treatments balanced for all essential amino acids were used. When necessary, amino acids were estimated after reformulating the diets [1]. * Estimated levels.

**Table 3:** Summary of the studies included in the meta-analysis to estimate changes in average daily gain and gain to feed in the finishing phase.

BW, kg	CP Level, %		SID Lys, %	Supplemental L-lysine ^1^, %	Additional Amino Acids ^2^	Reference ^3^
	Control	Lower				
55–102	15.9	13.6	0.74	0.27	Val	Hansen et al. [78]
54–98	15.9	13.6	0.74	0.27	Val	Nørgaard et al. [70]
62–97	13.1	12.1	0.65	0.26	Val	Tous et al. [79]
72–104	15.3	12.0	0.71	0.39	Val, Ile	Xie et al. [68] (Exp. 1)
89–114	14.0	12.0	0.73	0.35	Val, Ile	Qin et al. [17]
65–95	14.0	10.0	0.73	0.43	Val, Ile, Phe	Zhou et al. [69]
59–98	15.6	11.6	0.85	0.46	Val, Ile	Jiao et al. [80]
62–100	16.0	13.0	0.72	0.21	-	Li et al. [71]
94–118	13.7	10.5	0.70	0.41	Val, Ile	Ma et al. [81]
109–127	13.0	10.0	0.66	0.41	Val, Ile	Soto et al. [12] (Exp. 2)
112–135	13.0	9.0	0.55	0.34	Val, Ile	Soto et al. [12] (Exp. 3)
74–107	13.6	11.1	0.73	0.42	Val	Li et al. [82]
59–95	16.0	12.0	0.73	0.29	-	Zhou et al. [72]

^1^ Supplemented feed grade L-lysine based on the purity of L-lysine HCl or L-lysine sulfate in the lower-CP diet. ^2^ Additional supplemented feed grade amino acids in low-CP diets in addition to Lys, Met, Tre, and Trp. ^3^ References listed in year order, only studies and treatments balanced for all essential amino acids were used. When necessary estimated after reformulating the diets [1].

## Data Availability

Publicly available datasets were analyzed in this study. This data can be found here: https://www.ncbi.nlm.nih.gov/.

## References

[1] National Research Council (2012). NRC Nutrient Requirements of Swine.

[2] Kim S.W., Hansen J.A., Chiba L.I. (2022). Diet Formulation and Feeding Programs. Sustainable Swine Nutrition.

[3] Rostagno H.S.S., Albino L.F.T., Donzele J.L., Gomes P.C., de Oliveira R.F., Lopes D.C., Ferreira A.S., Barreto S.L.T., Euclides R. (2017). Brazilian Tables for Poultry and Swine.

[4] van Milgen J., Dourmad J.-Y. (2015). Concept and application of ideal protein for pigs. J. Anim. Sci. Biotechnol..

[5] Chung T.K., Baker D.H. (1992). Ideal amino acid pattern for 10-kilogram pigs. J. Anim. Sci..

[6] Millet S., Aluwé M., De Boever J., De Witte B., Douidah L., Van den Broeke A., Leen F., De Cuyper C., Ampe B., De Campeneere S. (2018). The effect of crude protein reduction on performance and nitrogen metabolism in piglets (four to nine weeks of age) fed two dietary lysine levels. J. Anim. Sci..

[7] Figueroa J.L., Lewis A.J., Miller P.S., Fischer R.L., Diedrichsen R.M. (2003). Growth, carcass traits, and plasma amino acid concentrations of gilts fed low-protein diets supplemented with amino acids including histidine, isoleucine, and valine. J. Anim. Sci..

[8] Che L.Q., Peng X., Hu L., Wu C., Xu Q., Fang Z.F., Lin Y., Xu S.Y., Li J., Feng B. (2017). The addition of protein-bound amino acids in low-protein diets improves the metabolic and immunological characteristics in fifteen- to thirty-five-kg pigs. J. Anim. Sci..

[9] Limbach J.R., Espinosa C.D., Perez-Calvo E., Stein H.H. (2021). Effect of dietary crude protein level on growth performance, blood characteristics, and indicators of intestinal health in weanling pigs. J. Anim. Sci..

[10] Rochell S.J., Alexander L.S., Rocha G.C., Van Alstine W.G., Boyd R.D., Pettigrew J.E., Dilger R.N. (2015). Effects of dietary soybean meal concentration on growth and immune response of pigs infected with porcine reproductive and respiratory syndrome virus. J. Anim. Sci..

[11] Peng X., Hu L., Liu Y., Yan C., Fang Z.F., Lin Y., Xu S.Y., Li J., Wu C.M., Chen D.W. (2016). Effects of low-protein diets supplemented with indispensable amino acids on growth performance, intestinal morphology and immunological parameters in 13 to 35 kg pigs. Animal.

[12] Soto J.A., Tokach M.D., Dritz S.S., Woodworth J.C., Derouchey J.M., Goodband R.D., Wu F. (2019). Optimal dietary standardized ileal digestible lysine and crude protein concentration for growth and carcass performance in finishing pigs weighing greater than 100 kg. J. Anim. Sci..

[13] Carpenter D.A., O’Mara F.P., O’Doherty J.V. (2004). The effect of dietary crude protein concentration on growth performance, carcass composition and nitrogen excretion in entire grower-finisher pigs. Irish J. Agric. Food Res..

[14] Zhao Y., Tian G., Chen D., Zheng P., Yu J., He J., Mao X., Yu B. (2019). Effects of varying levels of dietary protein and net energy on growth performance, nitrogen balance and faecal characteristics of growingfinishing pigs. Rev. Bras. Zootec..

[15] Wang H., Long W., Chadwick D., Velthof G.L., Oenema O., Ma W., Wang J., Qin W., Hou Y., Zhang F. (2020). Can dietary manipulations improve the productivity of pigs with lower environmental and economic cost? A global meta-analysis. Agric. Ecosyst. Environ..

[16] Moughan P.J., Fuller M.F. (2003). Modelling amino acid metabolism and the estimation of amino acid requirements. Amino Acids in Animal Nutrition.

[17] Qin C., Huang P., Qiu K., Sun W., Xu L., Zhang X., Yin J. (2015). Influences of dietary protein sources and crude protein levels on intracellular free amino acid profile in the longissimus dorsi muscle of finishing gilts. J. Anim. Sci. Biotechnol..

[18] Esteves L.A.C., Monteiro A.N.T.R., Sitanaka N.Y., Oliveira P.C., Castilha L.D., Paula V.R.C., Pozza P.C. (2021). The reduction of crude protein with the supplementation of amino acids in the diet reduces the environmental impact of growing pigs production evaluated through life cycle assessment. Sustainability.

[19] Batson K.L., Calderón H.I., Tokach M.D., Woodworth J.C., Goodband R.D., Dritz S.S., DeRouchey J.M. (2021). Effects of feeding diets containing low crude protein and coarse wheat bran as alternatives to zinc oxide in nursery pig diets. J. Anim. Sci..

[20] St-Pierre N.R. (2001). Invited review. Integrating quantitative findings from multiple studies using mixed model methodology. J. Dairy Sci..

[21] Jang K.B., Kim S.W. (2022). Role of milk carbohydrates in intestinal health of nursery pigs: A review. J. Anim. Sci. Biotechnol..

[22] Quigley J.D., Dennis T.S., Suarez-Mena F.X., Hill T.M., Aragona K.M. (2021). Meta-analysis of effects of age on intestinal digestibility of liquid feeds in young calves. JDS Commun..

[23] Nyachoti C.M., Omogbenigun F.O., Rademacher M., Blank G. (2006). Performance responses and indicators of gastrointestinal health in early-weaned pigs fed low-protein amino acid-supplemented diets. J. Anim. Sci..

[24] Deng D., Yao K., Chu W., Li T., Huang R., Yin Y., Liu Z., Zhang J., Wu G. (2009). Impaired translation initiation activation and reduced protein synthesis in weaned piglets fed a low-protein diet. J. Nutr. Biochem..

[25] Spring S., Premathilake H., DeSilva U., Shili C., Carter S., Pezeshki A. (2020). Low Protein-High Carbohydrate Diets Alter Energy Balance, Gut Microbiota Composition and Blood Metabolomics Profile in Young Pigs. Sci. Rep..

[26] Nørgaard J.V., Fernández J.A. (2009). Isoleucine and valine supplementation of crude protein-reduced diets for pigs aged 5–8 weeks. Anim. Feed Sci. Technol..

[27] Zheng L., Wei H., Cheng C., Xiang Q., Pang J., Peng J. (2016). Supplementation of branched-chain amino acids to a reduced-protein diet improves growth performance in piglets: Involvement of increased feed intake and direct muscle growth-promoting effect. Br. J. Nutr..

[28] Wu Y., Jiang Z., Zheng C., Wang L., Zhu C., Yang X., Wen X., Ma X. (2015). Effects of protein sources and levels in antibiotic-free diets on diarrhea, intestinal morphology, and expression of tight junctions in weaned piglets. Anim. Nutr..

[29] Duan Y., Duan Y., Li F., Li Y., Guo Q. (2016). Effects of supplementation with branched—chain amino acids to low—protein diets on expression of genes related to lipid metabolism in skeletal muscle of growing pigs. Amino Acids.

[30] Rattigan R., Sweeney T., Maher S., Ryan M.T., Thornton K., O’Doherty J.V. (2020). Effects of reducing dietary crude protein concentration and supplementation with either laminarin or zinc oxide on the growth performance and intestinal health of newly weaned pigs. Anim. Feed Sci. Technol..

[31] Zhao Y., Weaver A.C., Fellner V., Payne R.L., Kim S.W. (2014). Amino acid fortified diets for weanling pigs replacing fish meal and whey protein concentrate: Effects on growth, immune status, and gut health. J. Anim. Sci. Biotechnol..

[32] Cervantes-Pahm S.K., Stein H.H. (2010). Ileal digestibility of amino acids in conventional, fermented, and enzyme-treated soybean meal and in soy protein isolate, fish meal, and casein fed to weanling pigs. J. Anim. Sci..

[33] Deng Z., Duarte M.E., Jang K.B., Kim S.W. (2022). Soy protein concentrate replacing animal protein supplements and its impacts on intestinal immune status, intestinal oxidative stress status, nutrient digestibility, mucosa-associated microbiota, and growth performance of nursery pigs. J. Anim. Sci..

[34] Duarte M.E., Kim S.W. (2022). Intestinal microbiota and its interaction to intestinal health in nursery pigs. Anim. Nutr..

[35] Yue L.Y., Qiao S.Y. (2008). Effects of low-protein diets supplemented with crystalline amino acids on performance and intestinal development in piglets over the first 2 weeks after weaning. Livest. Sci..

[36] Gloaguen M., Le Floc’h N., Corrent E., Primot Y., van Milgen J. (2014). The use of free amino acids allows formulating very low crude protein diets for piglets. J. Anim. Sci..

[37] Zhang S., Qiao S., Ren M., Zeng X., Ma X., Wu Z., Thacker P., Wu G. (2013). Supplementation with branched-chain amino acids to a low-protein diet regulates intestinal expression of amino acid and peptide transporters in weanling pigs. Amino Acids.

[38] Kim S.W., Mateo R.D., Yin Y.-L., Wu G. (2006). Functional amino acids and fatty acids for enhancing production performance of sows and piglets. Asian-Australas. J. Anim. Sci..

[39] Kim S.W., Chen H., Parnsen W. (2019). Regulatory Role of Amino Acids in Pigs Fed on Protein-restricted Diets. Curr. Protein Pept. Sci..

[40] Wu G., Knabe D.A., Kim S.W. (2004). Arginine Nutrition in Neonatal Pigs. J. Nutr..

[41] Kim S.W., McPherson R.L., Wu G. (2004). Dietary Arginine Supplementation Enhances the Growth of Milk-Fed Young Pigs. J. Nutr..

[42] Zhan Z., Ou D., Piao X., Kim S.W., Liu Y., Wang J. (2008). Dietary arginine supplementation affects microvascular development in the small intestine of early-weaned pigs. J. Nutr..

[43] da Silva Gomes M., Valente Júnior D.T., de Oliveira Silva F.C., Cunha Júnior R.L., Ribeiro Junior V., Saraiva A., Rocha G.C. (2021). Effects of glutamine and glutamate on nursery piglets fed diets with different digestible lysine content. Semin. Ciências Agrárias.

[44] Valini G.A.C., Duarte M.S., Calderano A.A., Teixeira L.M., Rodrigues G.A., Fernandes K.M., Veroneze R., Serão N.V.L., Mantovani H.C., Rocha G.C. (2021). Dietary nucleotide supplementation as an alternative to in-feed antibiotics in weaned piglets. Animal.

[45] Hou L., Wang L., Qiu Y., Xiong Y., Xiao H., Yi H., Wen X., Lin Z., Wang Z., Yang X. (2021). Effects of protein restriction and subsequent realimentation on body composition, gut microbiota and metabolite profiles in weaned piglets. Animals.

[46] Lynegaard J.C., Kjeldsen N.J., Bache J.K., Weber N.R., Hansen C.F., Nielsen J.P., Amdi C. (2021). Low protein diets without medicinal zinc oxide for weaned pigs reduced diarrhoea treatments and average daily gain. Animal.

[47] Duan Y., Guo Q., Wen C., Wang W., Li Y., Tan B., Li F., Yin Y. (2016). Free Amino Acid Profile and Expression of Genes Implicated in Protein Metabolism in Skeletal Muscle of Growing Pigs Fed Low-Protein Diets Supplemented with Branched-Chain Amino Acids. J. Agric. Food Chem..

[48] da Cunha Valini G.A., de Souza Duarte M., de Amorim Rodrigues G., Veroneze R., Saraiva A., Hausman G., Rocha G.C. (2020). Guanidinoacetic acid supplementation on growth performance and molecular mechanisms of lean mass gain in nursery pigs. Cienc. Rural.

[49] Cho J.H., Chen Y.J., Min B.J., Yoo J.S., Wang Y., Kim I.H. (2008). Effects of reducing dietary crude protein on growth performance, odor gas emission from manure and blood urea nitrogen and IGF-1 concentrations of serum in nursery pigs. Anim. Sci. J..

[50] Toledo J.B., Furlan A.C., Pozza P.C., Piano L.M., Carvalho P.L.O., Peñuela-Sierra L.M., Huepa L.M.D. (2014). Effect of the reduction of the crude protein content of diets supplemented with essential amino acids on the performance of piglets weighing 6–15 kg. Livest. Sci..

[51] Zhou H., Chen D., Mao X., He J., Yu J., Zheng P., Luo J., Gao J., Htoo J.K., Yu B. (2019). Evaluation of standardized ileal digestible lysine requirement for 8–20 kg pigs fed low crude protein diets. Anim. Sci. J..

[52] Tang W., Qian Y., Yu B., Zhang T., Gao J., He J., Huang Z., Zheng P., Mao X., Luo J. (2019). Effects of Bacillus subtilis DSM32315 supplementation and dietary crude protein level on performance, gut barrier function and microbiota profile in weaned piglets. J. Anim. Sci..

[53] Liu H., Wu L., Han H., Li Y., Wang L., Yin J., Fan W., Bai M., Yao J., Huang X. (2019). Reduced dietary nitrogen with a high Lys:CP ratio restricted dietary N excretion without negatively affecting weaned piglets. Anim. Nutr..

[54] PIC, PIC (Pig Improvement Company) (2022). PIC Nutrition and Feeding Guidelines.

[55] Madrid J., Martínez S., López C., Orengo J., López M.J., Hernández F. (2013). Effects of low protein diets on growth performance, carcass traits and ammonia emission of barrows and gilts. Anim. Prod. Sci..

[56] Qiu K., Zhang X., Jiao N., Xu D., Huang C., Wang Y., Yin J. (2018). Dietary protein level affects nutrient digestibility and ileal microbiota structure in growing pigs. Anim. Sci. J..

[57] Powell S., Bidner T.D., Payne R.L., Southern L.L. (2011). Growth performance of 20- to 50-kilogram pigs fed low-crude-protein diets supplemented with histidine, cystine, glycine, glutamic acid, or arginine. J. Anim. Sci..

[58] Li Y., Yin J., Han H., Liu G., Deng D., Kim S.W., Wu G., Li T., Yin Y. (2018). Metabolic and Proteomic Responses to Long-Term Protein Restriction in a Pig Model. J. Agric. Food Chem..

[59] Roux M.L., Donsbough A.L., Waguespack A.M., Powell S., Bidner T.D., Payne R.L., Southern L.L. (2011). Maximizing the use of supplemental amino acids in corn-soybean meal diets for 20- to 45-kilogram pigs. J. Anim. Sci..

[60] Sotak-Peper K.M., Gonzalez-Vega J.C., Stein H.H. (2015). Concentrations of digestible, metabolizable, and net energy in soybean meal produced in different areas of the United States and fed to pigs. J. Anim. Sci..

[61] Lopez D.A., Lagos L.V., Stein H.H. (2020). Digestible and metabolizable energy in soybean meal sourced from different countries and fed to pigs. Anim. Feed Sci. Technol..

[62] Wolp R.C., Rodrigues N.E.B., Zangeronimo M.G., Cantarelli V.S., Fialho E.T., Philomeno R., Alvarenga R.R., Rocha L.F. (2012). Soybean oil and crude protein levels for growing pigs kept under heat stress conditions. Livest. Sci..

[63] Toledo J.B., Furlan A.C., Pozza P.C., Carraro J., Moresco G., Ferreira S.L., Gallego A.G. (2014). Reduction of the crude protein content of diets supplemented with essential amino acids for piglets weighing 15 to 30 kilograms. Rev. Bras. Zootec..

[64] Morales A., Buenabad L., Castillo G., Arce N., Araiza B.A., Htoo J.K., Cervantes M. (2015). Low-protein amino acid-supplemented diets for growing pigs: Effect on expression of amino acid transporters, serum concentration, performance, and carcass composition. J. Anim. Sci..

[65] Fang L.H., Jin Y.H., Do S.H., Hong J.S., Kim B.O., Han T.H., Kim Y.Y. (2019). Effects of dietary energy and crude protein levels on growth performance, blood profiles, and carcass traits in growing-finishing pigs. J. Anim. Sci. Technol..

[66] Wang Y.M., Yu H.T., Zhou J.Y., Zeng X.F., Wang G., Cai S., Huang S., Zhu Z.P., Tan J.J., Johnston L.J. (2019). Effects of feeding growing-finishing pigs with low crude protein diets on growth performance, carcass characteristics, meat quality and nutrient digestibility in different areas of China. Anim. Feed Sci. Technol..

[67] Zhao Y., Tian G., Chen D., Zheng P., Yu J., He J., Mao X., Huang Z., Luo Y., Luo J. (2019). Effect of different dietary protein levels and amino acids supplementation patterns on growth performance, carcass characteristics and nitrogen excretion in growing-finishing pigs. J. Anim. Sci. Biotechnol..

[68] Xie C.Y., Zhang G.J., Zhang F.R., Zhang S.H., Zeng X.F., Thacker P.A., Qiao S.Y. (2014). Estimation of the optimal ratio of standardized ileal digestible tryptophan to lysine for finishing barrows fed low protein diets supplemented with crystalline amino acids. Czech J. Anim. Sci..

[69] Zhou P., Zhang L., Li J., Luo Y., Zhang B., Xing S., Zhu Y., Sun H., Gao F., Zhou G. (2015). Effects of dietary crude protein levels and cysteamine supplementation on protein synthetic and degradative signaling in skeletal muscle of finishing pigs. PLoS ONE.

[70] Nørgaard J.V., Hansen M.J., Soumeh E.A., Adamsen A.P.S., Poulsen H.D. (2014). Effect of protein level on performance, nitrogen utilisation and carcass composition in finisher pigs. Acta Agric. Scand. A Anim. Sci..

[71] Li Y.H., Li F.N., Duan Y.H., Guo Q.P., Wen C.Y., Wang W.L., Huang X.G., Yin Y.L. (2018). Low-protein diet improves meat quality of growing and finishing pigs through changing lipid metabolism, fiber characteristics, and free amino acid profile of the muscle. J. Anim. Sci..

[72] Zhou X., Liu Y., Zhang L., Kong X., Li F. (2021). Serine-to-glycine ratios in low-protein diets regulate intramuscular fat by affecting lipid metabolism and myofiber type transition in the skeletal muscle of growing-finishing pigs. Anim. Nutr..

[73] Hou Y., Wu Z., Dai Z., Wang G., Wu G. (2017). Protein hydrolysates in animal nutrition: Industrial production, bioactive peptides, and functional significance. J. Anim. Sci. Biotechnol..

[74] Gardner M.L.G., Wood D. (1989). Transport of peptides across the gastrointestinal tract. Biochem. Soc. Trans..

[75] Sun X., Acquah C., Aluko R.E., Udenigwe C.C. (2020). Considering food matrix and gastrointestinal effects in enhancing bioactive peptide absorption and bioavailability. J. Funct. Foods.

[76] Nagasawa M., Murakami T., Sato M., Takahata Y., Morimatsu F., Furuse M. (2012). Dietary animal proteins alter monoamine metabolism in the brain. Anim. Sci. J..

[77] Le Dinh P., van der Peet-Schwering C.M.C., Ogink N.W.M., Aarnink A.J.A. (2022). Correction: Le Dinh et al. Effect of Diet Composition on Excreta Composition and Ammonia Emissions from Growing-Finishing Pigs. Animals **2022**, *12*, 229. Animals.

[78] Hansen M.J., Nørgaard J.V., Adamsen A.P.S., Poulsen H.D. (2014). Effect of reduced crude protein on ammonia, methane, and chemical odorants emitted from pig houses. Livest. Sci..

[79] Tous N., Lizardo R., Vilà B., Gispert M., Font-i-Furnols M., Esteve-Garcia E. (2014). Effect of reducing dietary protein and lysine on growth performance, carcass characteristics, intramuscular fat, and fatty acid profile of finishing barrows. J. Anim. Sci..

[80] Jiao X., Ma W., Chen Y., Li Z. (2016). Effects of amino acids supplementation in low crude protein diets on growth performance, carcass traits and serum parameters in finishing gilts. Anim. Sci. J..

[81] Ma W., Mao P., Guo L., Qiao S. (2020). Crystalline amino acids supplementation improves the performance and carcass traits in late-finishing gilts fed low-protein diets. Anim. Sci. J..

[82] Li P.L., Wu F., Wang Y.M., Wang C.P., Zhou J.Y., Zeng X.F., Qiao S.Y. (2021). Combination of non-protein nitrogen and N-carbamylglutamate supplementation improves growth performance, nutrient digestibility and carcass characteristics in finishing pigs fed low protein diets. Anim. Feed Sci. Technol..

